# Contribution of coherent motion to the perception of biological motion among persons with Schizophrenia

**DOI:** 10.3389/fpsyg.2013.00507

**Published:** 2013-08-13

**Authors:** Justine M. Y. Spencer, Allison B. Sekuler, Patrick J. Bennett, Bruce K. Christensen

**Affiliations:** ^1^Department of Psychology, Neuroscience and Behaviour, McMaster UniversityHamilton, ON, Canada; ^2^Department of Psychiatry and Behavioural Neurosciences, McMaster UniversityHamilton, ON, Canada

**Keywords:** biological motion, schizophrenia, paranoid, motion, global mechanisms, local mechanisms, perception

## Abstract

People with schizophrenia (SCZ) are impaired in several domains of visual processing, including the discrimination and detection of biological motion. However, the mechanisms underlying SCZ-related biological motion processing deficits are unknown. Moreover, whether these impairments are specific to biological motion or represent a more widespread visual motion processing deficit is unclear. In the current study, three experiments were conducted to investigate the contribution of global coherent motion processing to biological motion perception among patients with SCZ. In Experiments 1 and 2, participants with SCZ (*n* = 33) and healthy controls (*n* = 33) were asked to discriminate the direction of motion from upright and inverted point-light walkers in the presence and absence of a noise mask. Additionally, participants discriminated the direction of non-biological global coherent motion. In Experiment 3, participants discriminated the direction of motion from upright scrambled walkers (which contained only local motion information) and upright random position walkers (which contained only global form information). Consistent with previous research, results from Experiment 1 and 2 showed that people with SCZ exhibited deficits in the direction discrimination of point-light walkers; however, this impairment was accounted for by decreased performance in the coherent motion control task. Furthermore, results from Experiment 3 demonstrated similar performance in the discrimination of scrambled and random position point-light walkers.

## INTRODUCTION

Since Johansson (1973) first introduced point-light walkers as an experimental tool for examining the perception of human motion, many studies have demonstrated the sensitivity of the human visual system with respect to detecting and perceiving biological motion. For example, healthy individuals are able to recognize human actions from point-light stimuli following short presentation durations (e.g., 200 ms; Johansson, 1973), under conditions of additional dynamic noise (Bertenthal and Pinto, 1994), and when the number of illuminated joint markers has been substantially reduced (Johansson, 1973). Furthermore, infants as young as 3 months old are able to discriminate upright point-light walkers from inverted walkers (Bertenthal et al., 1984). Dynamic biological motion has also been shown to convey relevant social information, such as cues regarding a person’s affect (Atkinson et al., 2004) sex (Barclay et al., 1978; Pollick et al., 2005), intention to deceive (Runeson and Frykholm, 1983), and identity (Cutting and Kozlowski, 1977; Loula et al., 2005). As a result, biological motion can convey information not only about the perceptual characteristics (e.g., size and shape) of a walker, but also higher-order social information regarding a walker’s intentions and emotional states.

Individuals with schizophrenia (SCZ) exhibit deficits in several aspects of visual motion processing including speed discrimination (Chen et al., 1999; Clementz et al., 2007) and the perception of coherent global motion (Stuve et al., 1997; Chen et al., 2003). Furthermore, this population exhibits deficits in their ability to recognize and interpret social stimuli (Bigelow et al., 2006; Baas et al., 2008) or detect emotions from affective facial expressions (Edwards et al., 2001; Kohler et al., 2003; Johnston et al., 2006; Monkul et al., 2007). Given known deficits in both motion processing and social cognition, it has been suggested that people with SCZ also may be impaired on tasks of biological motion processing, and that such deficits may contribute to the above-noted social deficits. Indeed, several studies that have examined biological motion perception in SCZ suggest that these patients are impaired on biological motion processing tasks. Kim et al. (2005) demonstrated that people with SCZ show a deficit in recognizing biological motion activities compared to biological scrambled motion sequences. Moreover, people with SCZ performed similarly when completing a static global form detection task, suggesting that the observed group difference in the biological motion task was not due to a general performance deficit in SCZ patients. Additionally, Kim et al. (2011) found that people with SCZ were less able to detect and discriminate biological motion on tasks that included a noise mask and the perturbation of kinematic information, respectively.

Despite evidence suggesting that SCZ patients have greater difficulty detecting and discriminating biological motion, it is unclear whether these deficits are specific to biological motion *per se* or represent a more general deficit in perceiving global motion. Chen et al. (2003) measured direction discrimination thresholds using a sine wave grating, a task that requires only local motion processing, and random dot kinematograms, which requires global motion processing. Chen et al. (2003) found that SCZ patients had elevated direction discrimination thresholds only in the task that used random dot stimuli, which suggests that processing of global, but not local, motion is impaired in SCZ patients. Point-light walker stimuli contain both local and global cues: the trajectory of each dot constituting a point-light walker conveys information about the motion of a particular part of the human figure (e.g., the feet, the elbows, etc.), and grouping these local elements creates a holistic perception of the walker’s global form (e.g., a whole body). Moreover, previous research suggests that both local (Mather et al., 1992; Troje and Westhoff, 2006) and global processes (Bertenthal and Pinto, 1994; Beintema and Lappe, 2002; Pilz et al., 2010) contribute to the perception of point-light walkers. Hence, the results of Chen et al. (2003) raise the possibility that at least some of the SCZ-related deficit in biological motion tasks reflects a general deficit in global motion processing. According to this hypothesis, the effect of SCZ on the perception of biological motion should be diminished or eliminated once the general effect of SCZ on global motion processing is taken into account. The current experiments examine this hypothesis. To our knowledge, previous studies investigating biological motion processing in SCZ have not used control tasks that could estimate deficits in global, non-biological motion processing. For example, Kim et al. (2005) used a control task that measured the ability of participants to group stationary lines into a larger global form. Although this task considered the grouping of visual elements into a Gestalt, the use of static stimuli means that it does not provide an appropriate control for examining global motion deficits.

To investigate the contribution of global motion to biological motion perception, three experiments were completed in which participants were asked to discriminate the direction of motion from point-light walkers. In Experiment 1, we measured direction discrimination thresholds for upright and inverted point-light walkers embedded in a dynamic noise mask. Importantly, direction discrimination thresholds also were measured for non-biological global motion, consisting of coherently translating dots, embedded in a dynamic noise mask. To determine if the results of obtained in Experiment 1 generalize to supra-threshold conditions, Experiment 2 measured response accuracy in a direction discrimination task that used stimuli similar to those used in Experiment 1 but which did not contain dynamic noise. Finally, Experiment 3 investigated the contribution of local and global mechanisms to biological motion among participants with SCZ by using point-light walkers that contained only local motion information (scrambled point-light walkers) or global motion information (random position point-light walkers).

## GENERAL MATERIALS AND METHODS

### PARTICIPANTS

Thirty-three people with SCZ (5 female, 28 males) and 33 healthy controls (18 females, 15 males) participated in all experiments. All participants had normal or corrected-to-normal visual acuity, as ascertained using a standard Snellen chart, and reported an absence of lifetime neurological illness, brain injury, learning disability, current or past substance dependence, or medical conditions that could affect cognitive performance (e.g., coronary heart disease, type 1 diabetes).

All patients met criteria for SCZ (21 patients) or schizoaffective disorder (12 patients), as confirmed by the Mini International Neuropsychiatric Interview (M.I.N.I.; Sheehan et al., 1998), but did not meet criteria for any other Axis 1 disorder. Patients with SCZ were outpatients, medication-stable for at least the past 6 months, and were prescribed either typical (5 patients) or atypical antipsychotics (28 patients). Healthy control participants did not meet criteria for any Axis 1 disorder and were also excluded if they reported having a first-degree relative with a SCZ-spectrum illness. Estimates of Full Scale Intelligence Quotient (FSIQ) were obtained by prorating performance on the Matrix Reasoning and Information subtests from the Wechsler Adult Intelligence Scale, 3rd Edition (Wechsler, 1997). General cognitive functioning was assessed via the Repeatable Battery for the Assessment of Neuropsychological Status (Randolph, 1998). Patients with SCZ were also administered the Positive and Negative Syndrome Scale (PANSS; Kay et al., 1987) to assess current symptom status. In addition, both patients and controls were administered select scales from the Personality Assessment Inventory (PAI), including the Depression (Dep), Alcohol Problems (Alc), Drug Problems (Drg), Positive Impression Management (PIM), and Negative Impression Management (NIM) scales (Morey, 1991). Both groups were age-matched, but healthy controls had achieved significantly higher years of education. Regarding neuropsychological measures, significant differences were found across WAIS-III FSIQ and all RBANS indices. Although participants with SCZ also had significantly elevated scores on the PAI-Drg and PAI-Dep subscales compared to healthy controls, no single participant scored in a range suggesting significant clinical problems in these domains (i.e., *T* > 70). Moreover, no participant evidenced deliberate distortion of their responses across both validity scales (i.e., PIM and NIM). **Table 1** provides information characterizing the study participants. Ethics approval for the study was obtained by the St. Joseph’s Healthcare Hamilton Research Ethics Board. All participants provided written, voluntary consent to participate and received $10/hour for their participation. Each participant was tested in all three experiments. The order of the experiments was counterbalanced across participants.

**Table 1:** Means (SD) for demographic, neuropsychological, and clinical characteristics of the sample.

Variable	Healthy controls *n* = 33	SCZ *n* = 33
**Demographic**
Age (years)	38.76 (11.01)	42.78 (8.11)
Education (years)	15.24 (2.17)	13.00 (2.02)^*^
**Neuropsychological**
Estimated FSIQ	111.42 (13.72)	97.44 (16.43)^*^
RBANS	102.15 (16.14)	78.69(13.55)^*^
**Clinical**
PAI-Alc	46.36 (3.13)	49.50 (12.10)
PAI-Drg	50.18 (9.19)	56.47 (14.22)^*^
PAI-Dep	46.24 (7.24)	58.84 (13.27)^*^
PAI-PIM	53.76 (9.91)	52.72 (11.69)
PAI-NIM	45.58 (8.60)	51.19 (16.16)
PANSS-Pos	–	40.62 (4.23)
PANSS-Neg	–	42.34 (10.12)

* Indicates a significant difference between healthy controls and people with SCZ. FSIQ, Full Scale Intelligence Quotient; RBANS, Repeatable Battery for the Assessment of Neuropsychological Status; PAI, Personality Assessment Inventory; PANSS, Positive and Negative Syndrome Scale.

### APPARATUS AND STIMULI

All experimental tasks were programmed and presented using a Macbook Pro laptop computer with MATLAB and the Psychophysics and Video ToolBox extensions (Brainard, 1997; Pelli, 1997). Stimuli were presented on a 19-in monitor with a resolution of 1024 × 864 pixels and a refresh rate of 60 Hz.

### PROCEDURE

In each experiment, participants were seated in a darkened room and viewed the stimuli at a distance of 60 cm with their heads stabilized by a chin rest. On each trial, the direction of motion was either toward the left or the right, and participants were asked to identify the direction of motion displayed by the stimulus by pressing a key on a standard QWERTY computer keyboard [i.e., “a” key (left) and “l” key (right)]. Stimulus durations were intermixed randomly across trials. Prior to the experiments, participants performed 10 practice trials for each stimulus type to familiarize themselves with the stimuli.

#### Experiment 1

**Methods.** Experiment 1 used upright and inverted point-light walkers, in addition to the non-biological motion control task. The stimulus used in the control task consisted of 11 dots that moved coherently to the left or right at a speed of 7°/s. Point-light walker stimuli were generated using a modified version of Cutting’s classic point-light walker algorithm (Cutting, 1978; Thornton et al., 1998). The walkers consisted of 11 dots (2 × 2 pixels) that simulated points on the head, shoulder, elbows, wrists, hip, knees, and ankles. The starting position of the stride cycle was chosen randomly on every trial, which prevented participants from recognizing the walker simply from the starting point or from a specific frame. The walker, which subtended 1.9 × 4.2°, did not move across the screen, but rather appeared to walk in place, as if on a treadmill. Inverted walkers were rotated by 180° so that they appeared to be walking on the ceiling. The walker stimuli consisted of 5, 15, 30, or 45 frames presented at 25 frames per second, resulting in a total presentation times of 0.2, 0.6, 1.2, and 1.8 s, respectively. These specific durations were chosen based on previous research suggesting that durations shorter than 0.2 s are insufficient for direction discrimination whereas increasing duration beyond 1.8 s does not improve performance (Pilz et al., 2010). One complete gait cycle was achieved after 40 frames, or 1.6 s.

All stimuli were occluded with a dynamic noise mask composed of an array of dots whose positions varied randomly on each stimulus frame. The point-light walkers, the dynamic noise mask, and the control stimulus, were all constructed with dots that were identical in size and contrast. All stimuli were presented on a black background and were centered on the middle of the screen. Dot luminance was 67.4 cd/m^2^ and the background luminance was less than 1 cd/m^2^. Trials in the conditions using upright walkers, inverted walkers, and drifting dots were randomly intermixed.

Direction discrimination thresholds were estimated by varying the number of dots presented in the dynamic noise mask using a 3-up/1-down staircase procedure. Note that the staircase increased the number of mask dots (i.e., reduced the signal-to-noise ratio) after three consecutive correct responses, and decreased the number of mask dots (i.e., increased the signal-to-noise ratio) after one incorrect response. The staircase converged on the number of mask dots needed to produce 79% correct responses, which in this task corresponds to a *d’* of 1.14. Thresholds for each stimulus duration were estimated by averaging the last 10 reversals.

**FIGURE 1:**
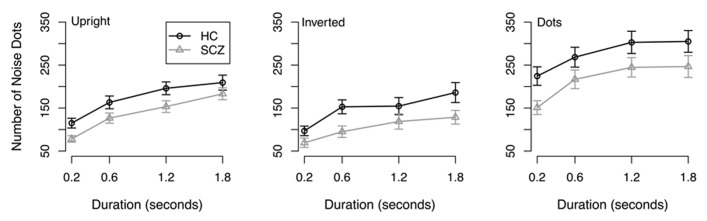
**Direction discrimination noise threshold for SCZ and healthy controls in the three conditions and across all durations.** Threshold is expressed as the number of dots in a dynamic noise mask; the point-light walker consisted of 11 dots. Both groups showed better performance in the dots condition compared to upright and inverted point-light walkers. Both groups also showed better performance in the upright condition compared to the inverted condition. No interactions were found.

**Results.** Direction discrimination thresholds are shown in **Figure 1**. Because thresholds are expressed in terms of the number of mask dots that are required to produce 79% correct responses, higher values correspond to better performance.

An initial 2 (group) × 3 (stimulus type) × 4 (stimulus duration) Analysis of Variance (ANOVA) was conducted on resulting thresholds. The ANOVA revealed a significant main effect of group (*F*(1, 64) = 4.89, *p* = 0.031), where participants with SCZ required fewer noise dots to reach threshold compared to healthy controls across all walker types and stimulus durations. A significant main effect of stimulus type was also observed (*F*(2, 128) = 34.85, *p* < 0.001) where both groups of participants demonstrated better performance in the coherent motion task compared to the upright (*t*(263) = 9.17, *p* < 0.001) and inverted point-light walker (*t*(263) = 11.42, *p* < 0.001) conditions. Bonferroni adjusted paired *t*-tests also revealed that participants performed significantly more accurately in the upright condition compared to the inverted condition (*t*(263) = 3.41, *p* < 0.001). A significant main effect of stimulus duration was also revealed: participants required fewer noise dots to reach threshold at 0.2 s duration, followed by 0.8, 1.2, and 1.6 s duration, respectively. All Bonferroni adjusted *t*-tests were significant (*p* < 0.05). No significant interactions were observed.

Results from the initial analysis suggest that people with SCZ have deficits in discriminating the direction of both biological motion and coherent non-biological motion. Importantly, the group × stimulus type interaction was not significant (*F*(2, 128) = 0.79, *p *= 0.45), which suggests that the difference between groups did not depend on the type of stimulus. Hence, the SCZ-related deficit in discriminating the direction of biological motion (i.e., point-light walkers) was no bigger than the deficit in discriminating non-biological motion (i.e., drifting dots). We therefore examined whether group differences in the upright and inverted walker conditions could be accounted for by performance in the control condition. First, we confirmed that the group difference in the upright and inverted walker conditions was significant: a 2 (group) × 2 (walker orientation) × 4 (stimulus duration) condition revealed significant main effects of group (*F*(1, 64) = 5.42, *p* = 0.023), walker orientation (*F*(1, 64) = 26.17, *p* < 0.001), and duration (*F*(3, 192) = 40.28, *p* < 0.001). The group × walker orientation interaction (*F*(1, 64) = 1.71, *p* = 0.19) was not significant, nor were any of the other interactions (in each case, *F* < 1.19, *p* > 0.31). To investigate whether group differences in biological motion discrimination can be accounted for by differences in global coherent motion discrimination, thresholds in the upright and inverted walker conditions were submitted to a 2 (group) × 2 (walker orientation) × 2 (stimulus duration) Analysis of Covariance (ANCOVA), where discrimination threshold in the control condition served as the covariate. In this analysis, the covariate was calculated by averaging thresholds in the control condition across stimulus duration, because a preliminary ANOVA on thresholds in the control condition failed to reveal a reliable group × stimulus duration interaction, (*F*(3, 192) = 2.10, *p* = 0.100), suggesting that group differences in the control condition did not vary as a function of stimulus duration. The ANCOVA revealed a significant effect of the covariate (*F*(1,63) = 34.68, *p *< 0.001), and significant main effects of walker orientation (*F*(1, 63) = 25.91, *p* < 0.001) and stimulus duration (*F*(3, 192) = 23.41, *p* < 0.001). Importantly, the main effect of group was not significant (*F*(1, 63) = 1.92, *p* = 0.171), nor were any of the interactions. These results suggest thresholds in the upright and inverted walker conditions do not differ between groups, once threshold in the control condition is taken into account.

#### Experiment 2

Consistent with previous reports (Kim et al., 2005, 2011), Experiment 1 found that people with SCZ exhibit deficits in the direction discrimination of biological motion. However, Experiment 1 also found that the group difference was not significant once threshold in a control task, which used non-biological global motion, was taken into account. This result suggests that deficits observed in the direction discrimination of point-light walkers among SCZ participants were not specific to biological motion but instead may represent more a more widespread processing deficit for global visual motion.

Experiment 1 measured direction discrimination thresholds, which means that our stimuli were presented at low signal-to-noise ratios. However, biological motion stimuli are presented at very high signal-to-noise ratios in many naturalistic contexts. Do SCZ patients exhibit biological motion processing deficits in such situations? Previous studies suggest that such deficits do exist: Kim et al. (2005), for example, used high signal-to-noise stimuli in a biological motion detection task and found that sensitivity in SCZ patients (*d’* = 2.2) was significantly lower than sensitivity in control participants (*d’* = 2.8). Also, Spencer et al. (2013) found that response accuracy in an emotion discrimination task using unmasked, high signal-to-noise ratio point-light walkers was significantly lower in SCZ patients than control subjects. However, neither one of these studies examined whether the SCZ-related deficits could be accounted for by deficits in non-biological, global motion processing. Therefore, Experiment 2 examined this question by having participants perform the same tasks as Experiment 1, but without the dynamic noise mask.

**Methods.** Experiment 2 used identical stimuli to those presented in Experiment 1, but with the dynamic noise mask removed.

**FIGURE 2:**
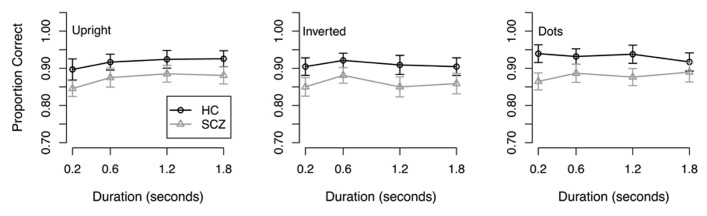
**Response accuracy from Experiment 2.** Both groups showed better performance in the dots condition compared to upright and inverted point-light walkers. Both groups also showed better performance in the upright condition compared to the inverted condition. No interactions were found.

**Results.** The dependant variable, proportion of correct responses, was not normally distributed; therefore, statistical analyses were performed on the arcsine-transformed data. First, transformed data were submitted to a 2 (group) × 3 (stimulus type) × 4 (stimulus duration) ANOVA. Results of this analysis revealed a significant main effect of group (*F*(1, 65) = 4.47, *p* = 0.038) where people with SCZ demonstrated reduced accuracy across all conditions and stimulus durations (**Figure 2**). A significant main effect of stimulus type was also observed (*F*(2, 130) = 9.19, *p* < 0.001) where both groups of participants performed more accurately in the coherent motion task compared to the inverted point-light walker condition (*t*(267) = 2.23, *p* = 0.026). Bonferroni corrected pairwise *t*-tests did not reveal significant differences between the other conditions. A main effect of stimulus duration was also revealed (*F*(3, 195) = 5.88, *p* < 0.001). Subsequent Bonferroni corrected *t*-tests revealed that significant differences were only found in the stimulus duration of 0.2 s (*p* < 0.05). Comparisons between 0.6, 1.2, and 1.8 s were not significantly different. The group × stimulus type interaction (*F*(2, 130) = 0.8, *p* = 0.45) was not significant, nor were any of the other interactions (*F* < 1.54, *p* > 0.20 in each case).

As in Experiment 1, we next conducted analyses to determine if the group differences in the upright and inverted walker conditions could be accounted for by differences in the control condition. First, we confirmed that the group difference was significant in the two walker conditions: a 2 (group) × 2 (walker orientation) × 4 (stimulus duration) ANOVA revealed significant main effects of walker orientation (*F*(1, 65) = 4.51, *p* = 0.037) and stimulus duration (*F*(3, 195) = 3.93, *p* = 0.01), and a significant walker orientation × stimulus duration interaction (*F*(3, 195) = 2.72, *p* = 0.046). The main effect of group approached significance (*F*(1, 65) = 3.75, *p* = 0.057), although a one-tailed test, which is appropriate for the prediction that accuracy is lower in the SCZ group, was significant (*t*(65) = 1.94, *p* = 0.028). Next, we analyzed the results in the control condition using a 2 (group) × 4 (stimulus duration) ANOVA: the main effect of group was significant (*F*(1, 65) = 5.69, *p* = 0.02) but the group × duration interaction was not (*F*(3, 192) = 1.83, *p* = 0.143), suggesting that the group difference did not vary as a function of stimulus duration. We therefore averaged performance across stimulus durations and used the resulting value as a covariate in a 2 (group) × 2 (walker orientation) × 4 (stimulus duration) ANCOVA. The ANCOVA revealed a significant effect of the covariate (*F*(1, 64) = 495.07, *p* < 0.001), significant main effects of both walker orientation (*F*(1, 64) = 4.47, *p* < 0.038) and stimulus duration (*F*(3, 192) = 3.99, *p* < 0.009), and a significant walker orientation × stimulus duration interaction (*F*(3, 189) = 2.70, *p* = 0.046). However, the main effect of group (*F*(1, 64) = 0.075, *p* = 0.389) was not significant, nor were any of the remaining interactions (*F* < 1.87, *p* > 0.13 in all cases). These results suggest that healthy controls and patients with SCZ perform similarly in the direction discrimination of supra-threshold biological motion stimuli once differences in coherent global motion are taken into account.

#### Experiment 3

The results of Experiments 1 and 2 suggest that SCZ-related deficits in the discrimination of the direction of point-light walkers is not specific to biological motion, but instead can be accounted for by more general global coherent motion processing deficits. This result was the same across both experiments, indicating that SCZ-related deficits in discriminating biological motion can be accounted for by global coherent motion processing regardless of signal-to-noise conditions.

To further investigate the contribution of global motion processing to biological motion discrimination, Experiment 3 compared SCZ and control group direction discrimination of scrambled and random position point-light walkers. In the *scrambled* condition, the trajectory of each local dot was maintained, but the initial dot positions were shifted randomly along the *x* and *y*-axes of the display, resulting in a point-light walker with intact local motion information but distorted global form (e.g., Thornton et al., 1998; Troje and Westhoff, 2006; Pilz et al., 2010). In the *random position* condition, each dot was shifted randomly between two adjacent joints across successive frames (e.g., Beintema and Lappe, 2002; Pilz et al., 2010), resulting in disrupted local trajectories of individual dots but preserved global form of a walker. A recent study by Pilz et al. (2010) using similar stimuli showed that among both younger and older adults, the removal of global elements from point-light walkers (i.e., scrambled point-light walkers) resulted in significantly reduced direction discrimination. Conversely, the removal of local motion information (i.e., random position point-light walkers) had little impact on performance. Given the results from Experiments 1 and 2 suggesting that SCZ-related deficits in biological motion discrimination can be accounted for by general deficits in global coherent motion processing, it was hypothesized that SCZ patients would be negatively impacted by the removal of global form information but undeterred by the removal of information regarding local position.

**Methods.** In Experiment 3, healthy controls and patients with SCZ discriminated the direction of upright, scrambled, and random position point-light walkers. As in the previous experiments, stimuli were presented in four durations (0.2, 0.8, 1.2, and 1.6 s), which were randomized on every trial. Participants completed 20 trials for each type of walker at each stimulus duration, resulting in a total of 240 trials. The dependent variable was the proportion of correct responses.

**FIGURE 3:**
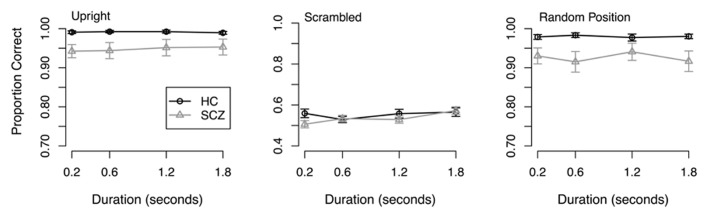
**Response accuracy from Experiment 3.** Both groups of participants showed better performance in the upright and random position conditions compared to the scrambled condition. No interactions were found.

**Results.** Response accuracy is plotted as a function of stimulus duration in **Figure 3**. A 2 (group) × 3 (stimulus type) × 4 (duration) ANOVA on arcsine-transformed data revealed a significant main effect of group (*F*(1,64) = 7.17, *p* = 0.009), where people with SCZ were less accurate overall compared to healthy controls. The ANOVA also found a significant main effect of stimulus type (*F*(2, 128) = 1002.03, *p* < 0.001), such that response accuracy in both groups was greater in the upright and random position conditions compared to the scrambled condition. No other significant main effects or interactions were observed.

**FIGURE 4:**
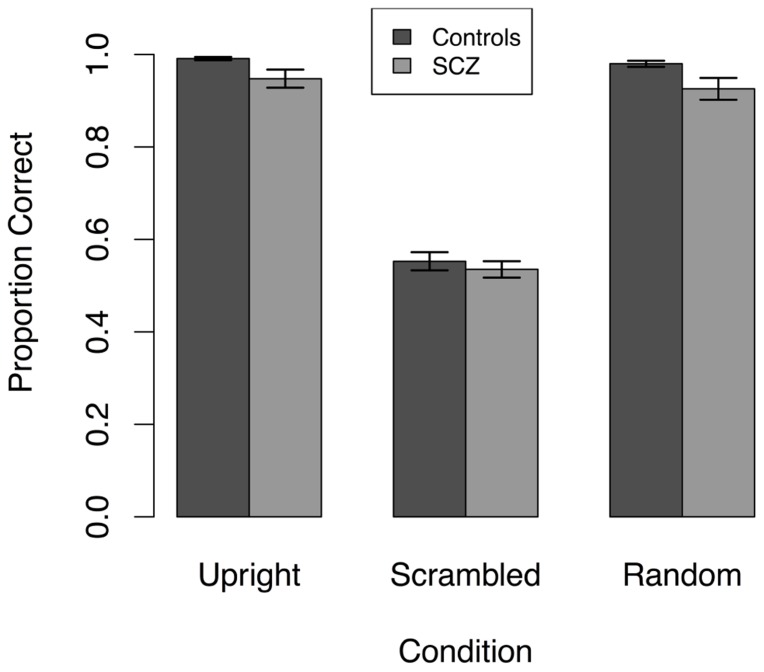
**Response accuracy averaged across stimulus durations in three conditions in Experiment 3.** Both SCZ and healthy controls performed significantly worse in the scrambled condition compared to the upright and random position conditions. Results shown are collapsed across all stimulus durations.

Because accuracy did not vary with stimulus duration, we averaged accuracy across stimulus duration for each participant. The mean of the averaged accuracy is plotted as a function of group and stimulus type in **Figure 4**, which illustrates that accuracy in both groups of participants was quite high in the upright and random walker conditions and near chance in the scrambled walker condition. Hence, manipulation of local and global information had qualitatively similar effects in SCZ patients and healthy controls.

However, **Figure 4** also highlights the existence of ceiling effects in the upright and random walker conditions and a possible floor effect in the scrambled condition. These ceiling and floor effects would make it difficult for an ANOVA to find group differences in performance, and therefore we analyzed the data in another way. Following an approach previously suggested by Kim et al. (2011), first, we compared the proportion of participants in each group whose accuracy in each of the upright and random conditions was less than 1.0, and whose accuracy in the scrambled condition was significantly greater than chance (i.e., accuracy ≥0.587, *p* < 0.05, one-tailed). In each condition, the proportions of participants in the SCZ and control groups did not differ (see **Table 2**). Next, we used *t*-tests to compare the mean response accuracy for participants in each group who had an accuracy less than 1.0 in the upright and random walker conditions and above chance in the scrambled condition: for these subsets of participants, accuracies in the SCZ group and the control group differed in the upright and random walker conditions, but not the scrambled walker condition (see **Table 3**).

**Table 2:** Proportion of participants in Experiment 3 with response accuracies that were less than 1.0 in the upright and random walker conditions and greater than chance in the scrambled walker condition.

	SCZ	Controls	Chi-square	*p*
Upright	17/33 = 0.51	16/33 = 0.49	0	0.99
Scrambled	6/33 = 0.18	13/33 = 0.39	2.66	0.10
Random	22/33 = 0.67	20/33 = 0.61	0.065	0.79

**Table 3:** Mean response accuracy of participants who had an accuracy less than 1.0 in the upright and random conditions and greater than chance in the scrambled condition.

	SCZ	Controls	*t*	*df*	*p*
Upright	0.898	0.982	2.58	31	0.02
Scrambled	0.606	0.613	0.62	17	0.54
Random	0.888	0.966	2.39	40	0.02

Taken together, our analyses suggest that there was a small group difference in response accuracy in the upright and random conditions, but not in the scrambled condition. However, the primary finding was that manipulations of local motion information and global form had similar effects on direction discrimination in both groups.

## GENERAL DISCUSSION

The present study examined the effects of SCZ on the perception of biological motion. Consistent with previous reports (Kim et al., 2005, 2011), Experiments 1 and 2 found that SCZ patients were worse than healthy control participants at discriminating the direction of upright and inverted point-light walkers. However, Experiments 1 and 2 also found that SCZ patients were worse in a control task that required participants to discriminate the direction of non-biological global motion (i.e., coherently drifting dots). Furthermore, we found that group differences in conditions that used point-light walkers were eliminated once performance in the control task was taken into account. Taken together, the results of Experiments 1 and 2 suggest that although people with SCZ do exhibit deficits in the direction discrimination of point-light walkers, this impairment is not specific to biological motion *per se* but likely represents more general deficits in global motion processing.

Experiment 3 also found evidence that SCZ patients were slightly less accurate at discriminating the direction of standard upright point-light walkers as well as random walkers that contained the global form, but not the local motion cues. These results suggest that patients with SCZ exhibit deficits utilizing global form compared to healthy controls. Although no significant group differences were found in the scrambled condition, it is more difficult to speculate regarding local motion mechanisms, as most participants in both groups were found to perform at chance level. Additionally, results of Experiment 3 demonstrate that manipulations of the global form and local motion information in point-light walkers had similar effects on direction discrimination in both patients with SCZ and control participants: in both groups, removing local motion cues (i.e., the random position condition) had small effects on performance, but removing global form cues (i.e., the scrambled condition) made discrimination much more difficult. This result suggests that the relative influence of global form and local motion cues on the perceived direction of point-light walkers is similar in people with SCZ and healthy controls. Despite these results, it is difficult to speak directly to the nature of local and global mechanisms in this experiment given the observed floor and ceiling effects (see **Figure 4**). The use of a noise mask to remove performance from the floor and ceiling would be helpful to examine specific contributions of local and global mechanisms to biological motion processing and represents an avenue of future study. Nevertheless, despite floor and ceiling effects, results from Experiment 3 demonstrate that people with SCZ performed similarly to that of healthy controls, in that form information is important for the direction discrimination of point-light walkers. Furthermore, these results are consistent with Pilz et al. (2010), in which removing global form from point-light walkers was shown to reduce performance in both younger and older healthy adults. As a result, using the identical mechanism to alter the point-light walker stimuli resulted in similar performances among healthy controls and people with SCZ, suggesting that the mechanisms used during this experiment were also likely similar.

It is also important to note that the stimuli used in the current study were generated using a modified version of Cutting’s classic point-light walker algorithm. Although these stimuli have been used repeatedly in previous studies, more recent research by Saunders et al. (2009) has suggested that the Cutting point-light walker algorithm lacks important visual information associated with the local motion of dots representing the feet compared to more naturalistic point-light walkers displays. Specifically, Saunders et al. (2009) show that while detection performance of Cutting and naturalistic point-light walkers was unchanged, participants were better able to discriminate the direction of motion from scrambled naturalistic point-light walkers compared to Cutting point-light walkers. Given these results, future research should utilize naturalistic point-light walkers to further examine deficits in SCZ regarding biological motion perception.

Regarding additional limitations, all patients with SCZ who took part in the current study were medicated, and we are unable to comment as to whether the results observed in the study were confounded by medication status. Additionally, analysis of sample characteristics revealed that the patients in the study had a significantly lower education level, as well as estimated intelligence levels and general neuropsychological scores. The issue of how to approach confounding group differences, however, is a complicated one and a topic of active debate within SCZ research. On the one hand, several characteristics that reliably distinguish SCZ from healthy participants are indeed correlated with outcomes of interest. In this situation, it is conventional to attempt to equate between-group differences on the confounding variable via linear covariate analyses. The popularity of these methods notwithstanding, they have been criticized on both statistical and conceptual grounds. In the case of the latter, Meehl (1970) has argued that if the confounding variable is a valid reflection of a pathological state (e.g., psychological symptoms), linear removal of the shared variance will necessarily attenuate the between-group variance of interest. Statistically, as Miller and Chapman (2001) and others discuss, the use of ANCOVA to correct for factors such as IQ is statistically dubious, as this analysis assumes that the covariate and independent variable, such as diagnostic group, are independent (Silverstein, 2008). As such, in psychopathological research, these variables are often not independent, and using ANCOVA to control for a covariate in psychopathology research removes meaningful variance from the independent variable of interest. Consequently, in the absence of random assignment, group membership is generally acknowledged to represent a broad collection of symptoms and problems that denote the entirety of a psychopathological category.

The perception of global coherent motion in random dot patterns requires the visual system to represent the speed and direction of individual dots and to integrate such information across space and time. Chen et al. (2003) presented evidence that processing of global, but not local, motion is impaired in SCZ, which suggests that spatiotemporal integration of local motion cues is deficient in SCZ patients. Given this apparent spatiotemporal motion integration deficit in patients with SCZ, and the fact that biological motion processing involves the integration of both local and global cues (Mather et al., 1992), it is not surprising that SCZ-related deficits in biological motion processing have been observed in previous studies (Kim et al., 2005, 2011). Deficits in global motion processing also are consistent with an fMRI (functional magnetic resonance imaging) study by Chen et al. (2008) which found that SCZ patients had reduced activation in middle temporal area (MT), a cortical area that has been implicated in global motion processing (Maunsell and Newsome, 1987), during tasks of coherent motion and speed discrimination, but not during a task of contrast discrimination. Interestingly, Chen et al. (2008) also found greater activation in prefrontal cortex during motion tasks in SCZ patients than control participants, suggesting that higher-order cognitive processes may be used by SCZ patients as a compensatory mechanism for motion processing deficits.

However, not all studies have found differential activation of MT in SCZ patients. Recently, Kim et al. (2011) reported that the overall pattern of brain activity associated with the processing of biological motion, but not activation in area MT, differs between healthy controls and patients with SCZ. Kim et al. (2011) interpreted their results as showing that deficits observed in biological motion among patients with SCZ were not solely attributable to motion processing more generally. One explanation for the lack of differential MT activity observed by Kim et al. (2011) is that behavioral differences in biological motion may instead involve mechanisms underlying the integration of spatial and temporal motion cues. People with SCZ exhibit deficits in spatial (Doniger et al., 2001, 2002) and temporal (Schwartz et al., 1983; Izawa and Yamamoto, 2002) integration. For example, compared to healthy controls, patients with SCZ are less able to spatially integrate fragmented images into coherent objects (Doniger et al., 2001). Furthermore, using event-related potential recordings, the inability to integrate these fragments has been correlated positively with dorsal stream processing in people with SCZ (Doniger et al., 2002), which is consistent with a wealth of literature suggesting impaired dorsal stream function in this population (Selemon et al., 1995; Gur et al., 2000; King et al., 2008).

Many studies have also implicated cortical area STSp (posterior superior temporal sulcus), a component of the dorsal stream network, in the perception of biological motion (e.g., Bondra et al., 1996; Grossman and Blake, 2001, 2002; Puce and Perrett, 2003). However, there is some evidence that the activity of the STSp differs in SCZ patients. For example, Kim et al. (2011) used fMRI to show that activation in STSp was higher when viewing biological motion than non-biological motion in healthy controls but not SCZ patients. Given that the STSp is a component of the dorsal stream network and also involved in the integration of other sensory information (Barraclough et al., 2005) the lack of STSp activity in response to biological motion among patients with SCZ may reflect a general integration deficit regarding visual elements. Furthermore, Giese and Poggio (2003) have argued that STS plays an important role in integrating information from the ventral and dorsal pathways into a single, coherent percept of biological motion. Given proposed integration deficits in people with SCZ, the differential activation of STSp reported by Kim et al. (2011) may reflect a more general impairment in the integration of visual elements.

In summary, the current experiments suggest that differences between SCZ and healthy controls in the ability to discriminate the direction of point-light walkers can be accounted for by SCZ-related deficits in the ability to perceive the direction of non-biological motion, which may be caused by deficits in spatial and/or temporal integration. However, in addition to having a perceived direction of motion, point-light walkers also can convey complex social information such as affect, intention, and identity. It is entirely possible that SCZ patients have deficits in perceiving this social information that cannot be accounted for by differences in spatial and temporal integration of non-biological motion. Given well-documented impaired social cognition among persons with SCZ (Hooker and Park, 2002; Bigelow et al., 2006; Monkul et al., 2007; Baas et al., 2008), it is important examine the perception of biological motion in its relation to social cognition.

## Conflict of Interest Statement

The authors declare that the research was conducted in the absence of any commercial or financial relationships that could be construed as a potential conflict of interest.

## References

[B1] Atkinson A. P. Dittrich W. H. Gemmell A. J. Young A. W. (2004) Emotion perception from dynamic and static body expressions in point-light and full-light displays. *Perception* 33 717–746 10.1068/p5096 15330366

[B2] Baas D. van’t Wout M. Aleman A. Khan R. S. (2008) Social judgement in clinically stable patients with schizophrenia and healthy relatives: behavioural evidence of social brain dysfunction. *Psychol. Med.* 38 747–754 10.1017/S0033291707001729 17988413

[B3] Barclay C. Cutting J. Kozlowski L. (1978) Temporal and spatial factors in gait perception that influence gender recognition. *Percept. Psychophys.* 23 145–152 10.3758/BF03208295 643509

[B4] Barraclough N. E. Xiao D. Baker C. I. Oram M. W. Perrett D. I. (2005) Integration of visual and auditory information by superior temporal sulcus neurons responsive to the sight of actions. *J. Cogn. Neurosci.* 17 377–391 10.1162/0898929053279586 15813999

[B5] Beintema J. Lappe M. (2002) Perception of biological motion without local image motion. *Proc. Natl. Acad. Sci. U.S.A.* 99 5661–5663 10.1073/pnas.082483699 11960019 PMC122827

[B6] Bertenthal B. I. Pinto J. (1994) Global processing of biological motions. *Psychol. Sci.* 5 221–225 10.1111/j.1467-9280.1994.tb00504.x

[B7] Bertenthal B. I. Proffitt D. R. Cutting J. E. (1984) Infant sensitivity to figural coherence in biomechanical motions. *J. Exp. Child Psychol.* 37 213–230 10.1016/0022-0965(84)90001-8 6726112

[B8] Bigelow N. O. Paradiso S. Adolphs R. Moser D. J. Arndt S. Heberlein A. (2006) Perception of socially relevant stimuli in schizophrenia. *Schizophr. Res.* 83 257–267 10.1016/j.schres.2005.12.856 16497483

[B9] Bondra E. Petrides M. Ostry D. Evans A. (1996) Specific involvement of human parietal systems and the amygdala in the perception of biological motion. *J. Neurosci.* 16 3737–3744 8642416 10.1523/JNEUROSCI.16-11-03737.1996 PMC6578830

[B10] Brainard D. H. (1997) The psychophysics toolbox. *Spat. Vis.* 10 443–446 10.1163/156856897X00357 9176952

[B11] Chen Y. Grossman E. D. Bidwell L. C. Yurgelun-Todd D. Gruber S. A. Levy D. L. (2008) Differential activation patterns of occipital and prefrontal cortices during motion processing: evidence from normal and schizophrenic brains. *Cogn. Affect. Behav. Neurosci.* 8 293–303 10.3758/CABN.8.3.293 18814466 PMC2572781

[B12] Chen Y. Nakayama K. Levy D. Matthysse S. Holzman P. (2003) Processing of global, but not local, motion direction is deficient in schizophrenia. *Schizophr. Res.* 61 215–227 10.1016/S0920-9964(02)00222-0 12729873

[B13] Chen Y. Nakayama K. Levy D. L. Matthysse S. Holzman P. S. (1999) Psychophysical isolation of a motion-processing deficit in schizophrenics and their relative and its association with impaired smooth pursuit. *Proc. Natl. Acad. Sci. U.S.A.* 96 4724–4729 10.1073/pnas.96.8.4724 10200329 PMC16399

[B14] Clementz B. A. McDowell J. E. Dobkins K. R. (2007) Compromised speed discrimination among schizophrenia patients when viewing smooth pursuit targets. *Schizophr. Res.* 95 61–65 10.1016/j.schres.2007.05.043 17628436 PMC3164492

[B15] Cutting J. E. (1978) A program to generate synthetic walkers as dynamic point-light displays. *Behav. Res. Methods Instrum. Comput.* 1 91–94 10.3758/BF03205105

[B16] Cutting J. E. Kozlowski L. T. (1977) Recognition of friends by their walk. *Bull. Psychon. Soc.* 9 353–356

[B17] Doniger G. M. Foxe J. J. Murray M. M. Higgins B. A. Javitt D. C. (2002) Impaired visual object recognition and dorsal/ventral stream interaction in schizophrenia. *Arch. Gen. Psychiatry* 59 1011–1020 10.1001/archpsyc.59.11.1011 12418934

[B18] Doniger G. M. Silipo G. Rabinowicz E. F. Snodgrass J. G. Javitt D. C. (2001) Impaired sensory processing as a basis for object-recognition deficits in schizophrenia. *Am. J. Psychiatry* 158 1818–1826 11691687 10.1176/appi.ajp.158.11.1818

[B19] Edwards J. Pattison P. Jackson H. Wales R. (2001) Facial affect and affective prosody recognition in first-episode schizophrenia. *Schizophr. Res.* 48 235–253 10.1016/S0920-9964(00)00099-2 11295377

[B20] Giese M. A. Poggio T. (2003) Neural mechanisms for the recognition of biological movements. *Nat. Rev. Neurosci.* 4 179–192 10.1038/nrn1057 12612631

[B21] Grossman E. D Blake R. (2001) Brain activity evoked by by inverted and imagined biological motion. *Vision Res.* 41 1475–1482 11322987 10.1016/s0042-6989(00)00317-5

[B22] Grossman E. D. Blake R. (2002) Brain areas active during visual perception of biological motion. *Neuron* 35 1167–1175 10.1016/S0896-6273(02)00897-8 12354405

[B23] Gur R. E. Cowell P. E. Latshaw A. Turetsky B. I. Grossman R. I. Arnold S. E. (2000) Reduced dorsal and orbital prefrontal gray matter volumes in schizophrenia. *Arch. Gen. Psychiatry* 57 761–768 10.1001/archpsyc.57.8.761 10920464

[B24] Hooker C. Park S. (2002) Emotion processing and its relationship to social functioning in schizophrenia patients. *Psychiatry Res.* 112 41–50 10.1016/S0165-1781(02)00177-4 12379449

[B25] Izawa R. Yamamoto S. (2002) Spatio-temporal disintegration of visual perception in schizophrenia as revealed by a novel cognitive task, the Searchlight Test. *Schizophr. Res.* 53 67–74 11728839 10.1016/s0920-9964(00)00116-x

[B26] Johansson G. (1973) Visual perception of biological motion and a model for its analysis. *Percept. Psychophys.* 14 195–204 10.3758/BF03212378

[B27] Johnston P. J. Devir H. Karayanidis F. (2006) Facial emotion processing in schizophrenia: no evidence for a deficit specific to negative emotions in a differential deficit design. *Psychiatry Res.* 143 51–61 10.1016/j.psychres.2005.08.006 16725209

[B28] Kay S. Fiszbein A. Opler L. (1987) The positive and negative syndrome scale (PANSS) for schizophrenia. *Schizophr. Bull.* 13 261–276 10.1093/schbul/13.2.261 3616518

[B29] Kim J. Doop M. L. Blake R. Park S. (2005) Impaired visual recognition of biological motion in schizophrenia. *Schizophr. Res.* 77 299–307 10.1016/j.schres.2005.04.006 15922565

[B30] Kim J. Park S. Blake R. (2011) Perception of biological motion in schizophrenia and healthy individuals: a behavioural and fMRI study. *PLoS ONE * 6:e19971 10.1371/journal.pone.0019971 PMC3098848 21625492

[B31] King J. P. Christensen B. K. Westwood D. A. (2008) Grasping behavior in schizophrenia suggests selective impairment in the dorsal visual pathway. *J. Abnorm. Psychol.* 117 799–811 10.1037/a0013500 19025227

[B32] Kohler C. G. Turner T. H. Bilker W. B. Brensinger C. M. Siegel S. J. Kanes S. J. (2003) Facial emotion intensity recognition in schizophrenia: intensity effects and error pattern. *Am. J. Psychiatry* 160 1768–1774 10.1176/appi.ajp.160.10.1768 14514489

[B33] Loula F. Prasad S. Harber K. Shiffrar M. (2005) Recognizing people from their movement. *J. Exp. Psychol. Hum. Percept. Perform.* 31 210–220 10.1037/0096-1523.31.1.210 15709874

[B34] Mather G. Radford K. West S. (1992) Low-level visual processing of biological motion. *Proc. Biol. Sci.* 249 149–155 10.1098/rspb.1992.0097 1360675

[B35] Maunsell J. H. R. Newsome W. T. (1987) Visual processing in monkey extrastriate cortex. *Annu. Rev. Neurosci.* 10 363–401 10.1146/annurev.ne.10.030187.002051 3105414

[B36] Meehl P. E. (1970) “Nuisance variables and the expostfacto design,” in *Minnesota Studies in the Philosophy of Science* eds Radner M. Winokur S. (Minneapolis: University of Minnesota Press) 373–402

[B37] Miller G. Chapman J. P. (2001) Misunderstanding analysis of covariance. *J. Abnorm. Psychol.* 110 40–48 10.1037/0021-843X.110.1.40 11261398

[B38] Monkul E. S. Green M. J. Barrett J. A. Robinson J. L. Velligan D. I. Glahn D. C. (2007) A social cognitive approach to emotional intensity judgment deficits in schizophrenia. *Schizophr. Res.* 94 245–252 10.1016/j.schres.2007.03.023 17583482

[B39] Morey L. C. (1991) *Personality Assessment Inventory*. Odessa: Psychological Assessment Resources, Inc

[B40] Pelli D. G. (1997) The VideoToolbox software for visual psychophysics: transforming numbers into movies. *Spat. Vis.* 10 437–442 10.1163/156856897X00366 9176953

[B41] Pilz K. S. Bennett P. J. Sekuler A. B. (2010) Effects of aging on biological motion discrimination. *Vision Res.* 50 211–219 10.1016/j.visres.2009.11.014 19941881

[B42] Pollick F. E. Kay J. W. Heim K. Stringer R. (2005) Gender recognition from point-light walkers. *J. Exp. Psychol. Hum. Percept. Perform.* 31 1247–1265 10.1037/0096-1523.31.6.1247 16366787

[B43] Puce A. Perrett D. I. (2003) Electrophysiology and brain imaging of biological motion. *Philos. Trans. R. Soc. Lond. B Biol. Sci.* 358 435–445 10.1098/rstb.2002.1221 12689371 PMC1693130

[B44] Randolph C. (1998) *Repeatable Battery for the Assessment of Neuropsychological Status (RBANS)*. San Antonio: Psychological Corporation

[B45] Runeson S. Frykholm G. (1983) Kinematic specification of dynamics as an informational bias for person-and-action perception: expectation, gender recognition, and deceptive intent. *J. Exp. Psychol. Gen.* 112 585–615 10.1037/0096-3445.112.4.585

[B46] Saunders D. R. Suchan J. Troje N. F. (2009) Off on the wrong foot: local features in biological motion. *Perception* 38 522–532 10.1068/p6140 19522321

[B47] Schwartz B. D. Winstead D. K Adinoff B. (1983) Temporal integration deficit in visual information processing by chronic schizophrenics. *Biol. Psychiatry* 18 1311–1320 6652164

[B48] Selemon L. D. Rajkowska G. Goldman-Rakic P. S. (1995) Abnormally high neuronal density in the schizophrenic cortex: a morphometric analysis of prefrontal area 9 and occipital area 17. *Arch. Gen. Psychiatry* 52 805–820 10.1001/archpsyc.1995.03950220015005 7575100

[B49] Sheehan D. V. Lecrubier Y. Sheehan K. H. Amorim P. Janavs J. Weiller E. (1998) The Mini-International Neuropsychiatric Interview (M.I.N.I.): the development and validation of a structured diagnostic psychiatric interview for DSM-IV and ICD-10. *J. Clin. Psychiatry* 59 22–33 9881538

[B50] Silverstein S. M. (2008) Measuring specific, rather than generalized, cognitive deficits and maximizing between-group effect size in studies of cognition and cognitive change. *Schizophr. Bull.* 34 645–655 10.1093/schbul/sbn032 18468987 PMC2632453

[B51] Spencer J. M. Y. Sekuler A. B. Bennett P. J. Giese M. A. Christensen B. K. (2013) Discriminating implicit and explicit emotions from point-light walkers in persons with schizophrenia. *Poster Presented at the Thirteenth Annual Vision Sciences Society Conference*, Naples.

[B52] Stuve T. A. Friedman L. Jesberger J. A. Gilmore G. C. Strauss M. E. Meltzer H. Y. (1997) The relationship between smooth pursuit performance, motion perception and sustained visual attention in patients with schizophrenia and normal controls. *Psychol. Med.* 23 143–152 10.1017/S0033291796004230 9122294

[B53] Thornton I. Pinto J. Shiffrar M. (1998) The visual perception of human locomotion. *Cogn. Neuropsychol.* 15 535–553 10.1080/026432998381014 22448837

[B54] Troje N. F. Westhoff C. (2006) The inversion effect in biological motion perception: evidence for a life detector? *Curr. Biol.* 16 821–824 10.1016/j.cub.2006.03.022 16631591

[B55] Wechsler D. (1997) *Wechsler Adult Intelligence Scale* 3rd Edn. San Antonio: The Psychological Corporation

